# A Model for an Angular Velocity-Tuned Motion Detector Accounting for Deviations in the Corridor-Centering Response of the Bee

**DOI:** 10.1371/journal.pcbi.1004887

**Published:** 2016-05-05

**Authors:** Alex J. Cope, Chelsea Sabo, Kevin Gurney, Eleni Vasilaki, James A. R. Marshall

**Affiliations:** 1 Department of Computer Science, University of Sheffield, Sheffield, South Yorkshire, United Kingdom; 2 Sheffield Robotics, Sheffield, South Yorkshire, United Kingdom; 3 Department of Psychology, University of Sheffield, Sheffield, South Yorkshire, United Kingdom; Northeastern University, UNITED STATES

## Abstract

We present a novel neurally based model for estimating angular velocity (AV) in the bee brain, capable of quantitatively reproducing experimental observations of visual odometry and corridor-centering in free-flying honeybees, including previously unaccounted for manipulations of behaviour. The model is fitted using electrophysiological data, and tested using behavioural data. Based on our model we suggest that the AV response can be considered as an evolutionary extension to the optomotor response. The detector is tested behaviourally in silico with the corridor-centering paradigm, where bees navigate down a corridor with gratings (square wave or sinusoidal) on the walls. When combined with an existing flight control algorithm the detector reproduces the invariance of the average flight path to the spatial frequency and contrast of the gratings, including deviations from perfect centering behaviour as found in the real bee’s behaviour. In addition, the summed response of the detector to a unit distance movement along the corridor is constant for a large range of grating spatial frequencies, demonstrating that the detector can be used as a visual odometer.

## Author Summary

We are interested in how bees are capable of navigating complex environments. Experimental evidence shows that they use an estimate of the speed that patterns move across their compound eyes (angular velocity) to control their behaviour and avoid obstacles, however the brain circuitry used to extract this information is not understood. We have created a model that uses a small number of assumptions to demonstrate a plausible set of circuitry. Since bees only extract an estimate of angular velocity they show differences from the expected behaviour for perfect angular velocity detection, and our model reproduces these differences.

## Introduction

It has long been established that the honeybee *Apis mellifera* and other insects control aspects of their flight by estimating the angular velocity (AV, the number of degrees per second subtended by a movement across the visual field, independent of the spatial frequency of the stimulus) of the environment around them. Examples include; positioning themselves laterally while navigating a patterned corridor [1–3], estimating distance [2], and maintaining forward velocity [4, 5]. They are capable of this behaviour over a wide range of perceived AV up to several hundred degrees per second. In contrast, other aspects of flight control, such as opposing rotations of the visual field (the classic optomotor response, which is the insect behaviour analogous to the vestibular-ocular reflex in vertebrates) are controlled by detecting the temporal frequency (number of contrast edges per second, dependent upon the spatial frequency of the visual stimulus) of the visual motion [6–8].

Currently the only elementary motion detection circuits experimentally identified in the insect optic neuropils respond to temporal frequency, and use the Reichardt-Hassenstein correlation detector architecture [9–17] replicated across a retinotopic array. A sub-population of the descending neurons in the bee neck also show a response consistent with the summed output of this retinotopic array [8]. These circuits therefore indicate a complete sensori-motor pathway capable of reproducing a set of behaviours that vary with the spatial frequency of the visual stimulus used. This can explain the classic optomotor response, but not the many other aspects of flight control where behaviour remains largely invariant to spatial frequency. In addition, the identified detectors only respond up to temporal frequencies of 10–100Hz [7, 8, 15, 18], far lower than those experienced in behavioural experiments on honeybees [2]. Additional neural circuitry capable of calculating an estimation for AV must therefore be present.

Ibbotson [18] recorded from descending neurons in the ventral nerve cord of the honeybee and found two distinct responses to the presentation of moving grating patterns in the bee’s field of view. The first group consists of temporal frequency responses consistent with the output for Reichard-Hassenstein detectors, showing a characteristic peak in response at a temporal frequency of 10Hz [8]. The second group shows a monotonically increasing response as the AV of the stimulus increases up to 1000Hz, with most neurons recorded showing little variance in response to changes in the spatial frequency of the stimulus. These neurons are descending to the motor regions of the bee, thus the signals transmitted by them could be used to control flight behaviour. As such, we believe that they provide the best existing evidence of what the output of the AV estimating neural circuitry might be.

There is clearly a gap in understanding between the identified frequency-dependent retinotopic motion detection circuitry, and the largely frequency-independent descending neurons which estimate AV. The lack of evidence for AV detection at the retinotopic level indicates that the solution may be implemented elsewhere. In insects the passage of visual information proceeds from sensory to motor via several pathways, the simplest of which proceeds retinotopically through the optic neuropils of the lamina and medulla to the lobula (or lobula and lobula plate in flies), where retinotopy is lost through summation by the wide-field neurons over their retinotopic inputs, then on to the posterior protocerebrum before descending to the motor ganglia [19–24]. Since the AV estimating responses have been found in the descending neurons, and not in the retinotopic regions, it therefore seems most likely that the production of these responses is performed in between the lobula and descending neurons.

The temporal frequency detection used in the optomotor response, and identified in the retinotopic part of the insect brain, shares many properties with the AV tuned neurons. Both respond to motion in a single preferred direction [8, 18] and, at least for a subset of optomotor neurons, are largely invariant to the contrast of the stimulus [2, 7]. Simpler insect species that share the optomotor response with bees, such as *Drosophila melanogaster*, do not use AV for flight regulation to the same extent (there is no AV based control of *Drosophila* height regulation [25] when compared with the bee [26, 27]; but there is AV based *Drosophila* speed regulation [5]).

AV estimation could have evolved in several different ways. The two we concentrate on are a circuit evolved separate to the optomotor circuit (as in some proposed models [28, 29]), or a circuit evolved as an extension to the optomotor circuit (as we propose here). A separate AV estimation circuit would have to exist as columnar circuits through the retinotopic regions of the insect visual system, covering either some, or all, of the visual field. In contrast a circuit comprising an extension to the optomotor circuit could combine outputs summed across all or some of the visual field. Therefore each neuron in the former circuit must be replicated across retinotopic locations, while the latter requires only a single neuron for each element in the circuit.

The evolution of the AV response is difficult to determine, as it exists across orders separated by hundreds of millions of years of evolution, however there are reasons to favour the hypothesis of an AV estimating extension to the optomotor circuit over that of a separate AV estimating circuit. Firstly there are energetic considerations. A complete additional retinotopic circuit requires orders of magnitude more neurons than an extension to the optomotor circuitry, hence evolutionary fitness costs for the organism; fitness benefits from such an arrangement would need to be substantial to offset this cost [30, 31]. Secondly there is the complexity of adding a complete neural circuit as compared to an extension, which requires only a few additional neurons. Thirdly, combining the outputs from existing elementary motion detectors will reduce the amount of information transferred (as the same information is used by the optomotor and AV estimating systems) and since information transfer is metabolically costly [30, 32–34] this provides a less energetically costly solution, and it has been shown that information rather than function is the key limit on miniaturisation [35].

An extension to the optomotor response circuitry could be constructed if there exist two or more populations of Reichardt-Hassenstein detectors which responded maximally to different temporal frequencies, possible evidence for which has been found in in the locust [36]. It has been suggested that the ratio of two such populations can provide an estimation of AV [37].

We therefore propose a model for the AV response in the bee as an extension of the optomotor pathway, allowing different detectors to be constructed by drawing selectively from a pool of elementary motion detectors with varying response timescales (due to spatial or temporal differences) and calculating the desired final response by combining the selected responses. We use the architecture of the elementary motion detectors found in *Drosophila* [15], however the increased size of the neurons in the bee brain means that we are unable to use the parameterisation of those detectors [30]. As there is a paucity of data with which to constrain the neuron parameters in the bee brain, we use the simplest form of neuron model to avoid an abundance of free parameters, with our chosen neuron model only requiring a single time constant that determines the rate that inputs are integrated and also decay, while containing dynamics to represent the time evolution of the signals found in the biological system.

Despite the simple neuron model the connection architecture used is based on the circuits found in the biology [15–18], with two key features assumed. Firstly, that there are populations of elementary motion detectors with different peak temporal frequencies. Secondly, that the output of these populations is summed by different wide field neurons in the lobula, and the resultant summations are combined, likely in the posterior protocerebrum, to create an AV estimation which is passed to the ventral nerve cord, which Ibbotson recorded from [18]. We can therefore define a model with few free parameters, which nonetheless integrates the currently understood biology of the bee AV estimation circuit. The test of such a model is how well it is in agreement with the behaviour found in real bees, and for this reason we validate the performance of the model in a simulated environment against data taken from experiments conducted in the field with real bees.

## Results

### A biologically constrained model of angular velocity estimation

Before describing the model in detail it is important to first describe the method used to tune the free parameters of our model, as this procedure is devised to minimise the number of assumptions we must make in parameterising the model.

While we assume the Reichardt-Hassenstein architecture as the basis for AV estimation response, it is important to note that we do not assume the parameterisation of this architecture. Instead we determine the parameters for the Reichardt-Hassenstein architecture by fitting our complete model to the neural AV estimation response found by Ibbotson [18], and only following this fitting do we investigate what response characteristics these parameters produce in the Reichardt-Hassenstein detectors. If our model is consistent with our assertions these should match the responses of the optomotor circuit detectors without further tuning. This provides an initial test of the validity of the model, as by only fitting the output of the full model we remove any assumptions about the time constants from the optomotor response literature.

**Fig 1:**
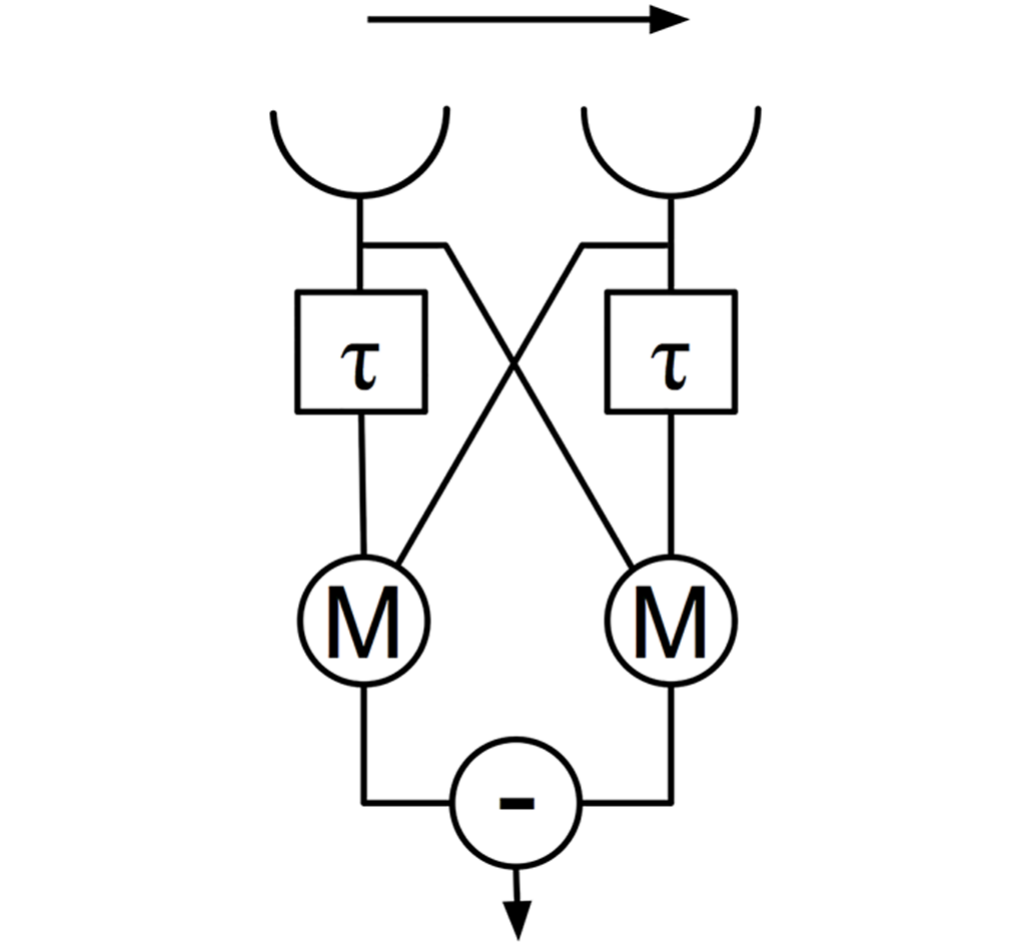
Reichardt-Hassenstein detector. The detector tests whether input at the two locations (top) is correlated in time, with peak response at the time constant *τ*. M represents multiplication, and—represents subtraction. By taking the difference between the progressive and regressive circuits (in this case the right arm is progressive and the left arm is regressive) the detector gives a response (bottom) from -I to +I, where I is the maximum input to the detector, and a negative value indicates a reverse correlation. The architecture of this detector forms the basis of the retinotopic layers of the model, however the form is modified by the addition of neural dynamics on both arms of the detector. Further details can be found in the text.

Each single detector unit in the full model contains biological implementations of the Reichardt-Hassenstein correlation detector (RHD) [11, 38] (the basic non-biologically implemented RHD detector is shown in Fig 1). These detector units are replicated in a retinotopic array representing the visual field of the bee across the array, and reproducing the columnar structure of the bee lamina and medulla from input to output of each detector unit. We hereafter will refer to an individual detector unit as an *Angular Velocity Detector Unit* (AVDU).

Each AVDU operates by responding to the coincidence between an event at one input location and the delayed copy of an event at another location. The two channels in the detector detect correlations in the progressive and regressive directions, and the difference gives an output between -1 and 1; respectively these outputs correspond to anti-correlation and correlation as measured in the progressive direction. The detector has a maximal response when the temporal difference between the events at the two locations is equal to the delay.

To recreate the response functions found in honeybee descending neurons we implement the suggestion of Zanker et al [37]. This involves considering the progressive and regressive arms of the RHD individually (see Fig 1). If the output of the regressive arm is subtracted from the output of the progressive arm with a scaling factor (*F*) of one, then the classical RHD described above is produced. Such a detector shows strong dependence on the stimulus spatial frequency and is unsuitable for calculating AV. If a value for *F* less than one is used then the spatial frequency dependence of the detector reduces with the value of *F* [37]. Zanker et al [37] suggest that the ratio of the outputs of the two half RHDs *(F = 0*) with different time constants can provide an invariant angular velocity response, but at the loss of directional tuning. We take this ratio following summation over the two detector arrays, assuming that the ratio therefore takes place after the lobula wide field neurons but before the descending neurons, most likely in the posterior protocerebrum of the bee. In addition to these modifications we also seek to create the detector in a biologically based manner, using rate-coded neural models.

**Fig 2:**
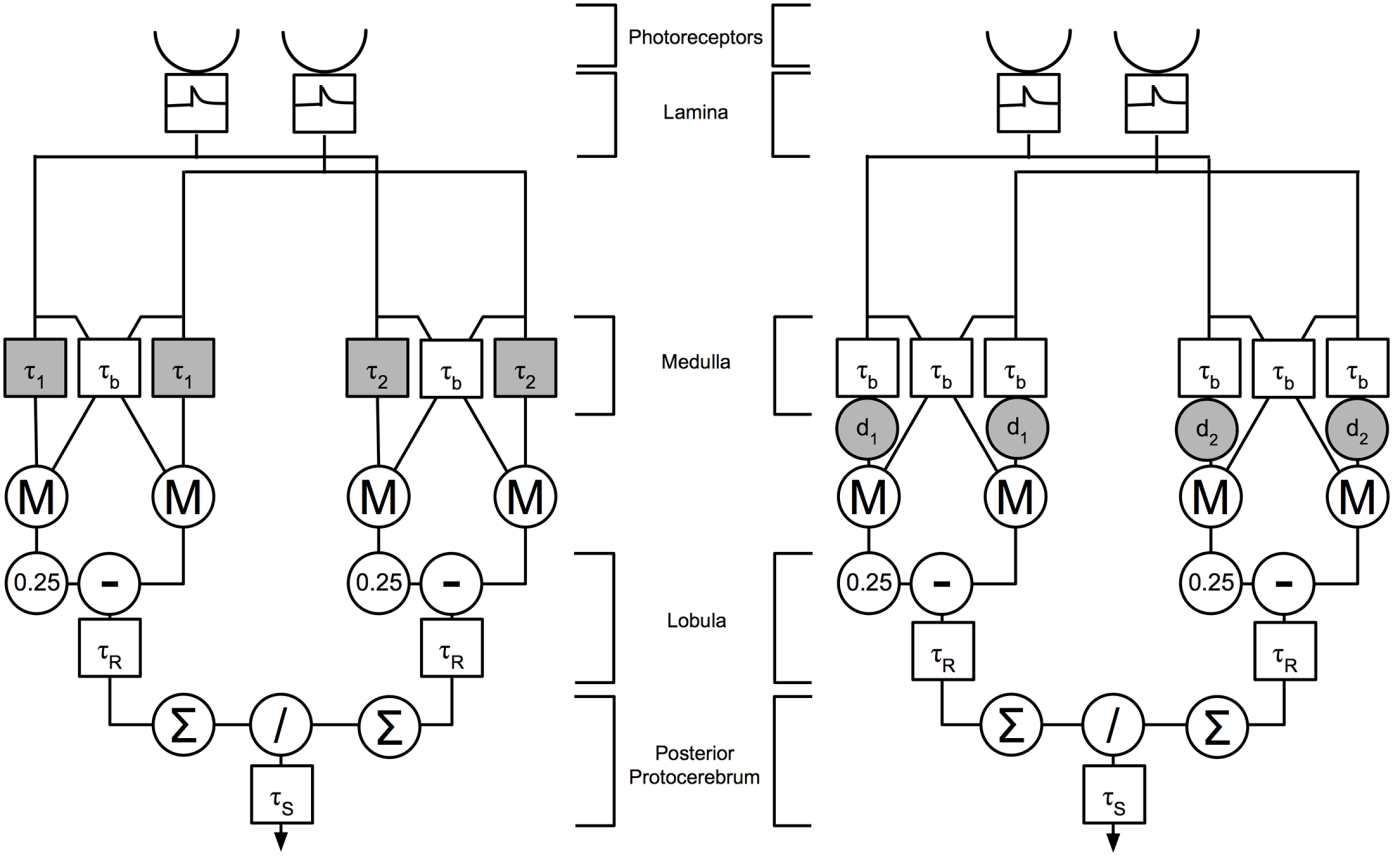
The two versions of the AVDU detector. Left: with dynamic time constants; right: with delays. Squares indicate LIN units, circles indicate other operations. In the centre we suggest the corresponding regions of the bee visual system for each stage of the detector. In both versions the input is first temporally filtered in the input layer (first square), then is transmitted to two Reichardt-Hassenstein detectors (Fig 1). These either differ in the time constants of the LINs (τ_1_ and τ_2_) or by fixed delays (d_1_ and d_2_) shown with gray backgrounds. A base time constant of τ_b_ = 1ms is used otherwise. A further LIN is used to apply the subtraction, having a time constant of τ_R_ = 5ms, and then the division is performed in the final LIN following summation across all detectors in the array. This final LIN has a time constant of τ_S_ = 100ms to smooth the output.

The structure of the model, including a biological implementation of the RHD (referred to here as the RHD-LIN—RHD-Leaky Integrator Neural unit) is shown in Fig 2. There are two types of square boxes (representing the neuron models) in the diagram, one with a t (which represents a rate-coded Leaky Integrator Neural unit (LIN)), the other without (denoting an adaptive-LIN).

Leaky Integrator Neural units (LINs) respond to their inputs *x*_i_ and update the internal activity *a* (analogous to the firing rate of the neuron) with a characteristic base time constant *τ_b_* as follows [39]:

$$\dot{a}=\frac{\left(-a+\sum x_{i}\right)}{\tau_{b}}.$$

The adaptive-LIN models the photoreceptors and first order neurons in the bee visual system. To mimic the fast adaptation found in the photoreceptors and laminar monopolar cells (LMC) [40, 41] of the bee these units consist of a simple slowly adapting activity level [39], with a separate adaptation current:

$${\dot{a}}_{PR}=\frac{\left(-a_{PR}-\alpha +x\right)}{\tau_{PR}},$$

$$\dot{\alpha }=\frac{\left(-\alpha +x\right)}{\tau_{\alpha }},$$

where *a*_PR_ is the output activity level of the photoreceptor, *x* the input, *τ*_PR_ (set to 8ms) a characteristic time constant, a the adaptation response, and τ_α_ (set to 15ms) the time constant of the adaptation response.

When testing this model one computational problem arises. In the ratio of the two RHD units there is an asymptote as the denominator tends to zero, and the output tends to very high values, which is undesirable and clearly not biologically plausible, as neurons have a maximum firing rate. In addition, many neurons have a tonic firing rate, and we use this to set a lower bound on the value of the denominator, preventing division by numbers close to zero. This bound is set at 0.01.

To quantify the behaviour of the detector, two versions are used: a test system to test the detector response curves to inputs, and a full system to test the model in a simulated virtual environment, data from which can then be compared to experimental data from real bees. These systems are detailed in the Methods.

### The neurons recorded by Ibbotson show log-linear responses to increasing AV

**Fig 3:**
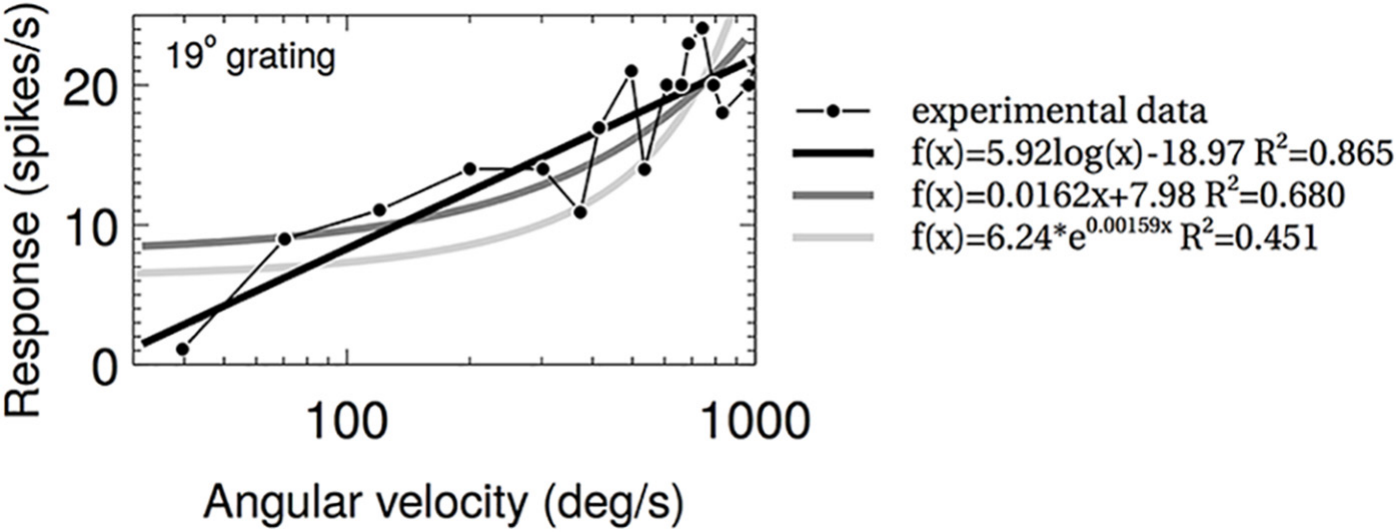
Example of fits to one data set from Ibbotson 2001. f(x) (response versus angular velocity) for log, linear and exponential fits are shown with their *R*^2^ values.

In order to tune our model to the response profiles of the AV estimating neurons recorded by Ibbotson [18] we must first characterise their responses, an extension we have undertaken to the analyses performed in the original paper. To do this we extracted data from the paper and fitted with linear, logarithmic and exponential functions (see Methods for details). The adjusted residuals for these fits are shown in the Supporting Information (S1 Appendices) and an example data fitting is shown in Fig 3. For the majority (8/13) of the data the best fit was provided by a logarithmic function, and the logarithmic fits had the highest average Adjusted *R*^2^ values of the three forms (0.7422 vs 0.6914 for linear and 0.6343 for exponential)—demonstrating that the log-linear relationship is a suitable choice. There were, however, several sets of data that were not best fitted by the logarithmic function, and instead were best fitted by a linear (1/13) or exponential (4/13) functions. Some data were poorly fitted by all forms, and this was due to the large variance in these data. A full table of all fitting values is presented in the Supplementary Material. The best fit to the data is therefore log-linear overall, however there is clearly some variation in the exact response profiles of the neurons. This variation, and possible explanations for it, will be explored later in the discussion, however for the fitting we will assume log-linear response.

### Model fitting I: Dynamical delays provide a better fit to the experimental data than fixed delays

To fit the model to the experimental data we first partitioned the data into one set for fitting (comprising the electrophysiological and anatomical data), and one set for testing (the behavioural data). In fitting the model to the electrophysiological data we examined the effect of two possible model design choices. First is the method of implementing the delay in the RHD-LINs. Second is the method of rectification from the adaptive-LIN.

It is possible for the delay in the RHD-LIN to be implemented in a biologically plausible manner in two different ways. First is a simple delay caused by differing axonal propagation speed [42], which will induce minimal distortion to the delayed signal. The second is delay due to the slow response of a neuron, whether by the use of a neurotransmitter with slow ion currents, or by slow dynamics in the membrane voltage of the whole neuron [43, 44]. This second mechanism necessarily distorts the delayed signal. In the first, all LINs have identical time constants and the delay in the RHD-LIN detectors are implemented as a fixed signal delay *d* (see Fig 2). In the second, the delay channel LIN has a time constant *τ* which is greater than *τ*_*b*_. It is likely that in a real biological system the delay would be composed of a combination of these two delay types, however given the short axonal distances in the bee brain it seems likely that the dynamical delays would dominate. We seek here to investigate whether such a hypothesis is consistent with the results from our model.

We investigated these two different variants of the model on the criteria identified in the previous result for the honeybee AV detector: a monotonically increasing log-linear response to increasing AV.

The form of the input rectification used in these experiments was half-rectified offset-only input (for details see the following section), as preliminary investigation showed that this method gives the best log-linear response—this assertion is justified in the following section.

**Fig 4:**
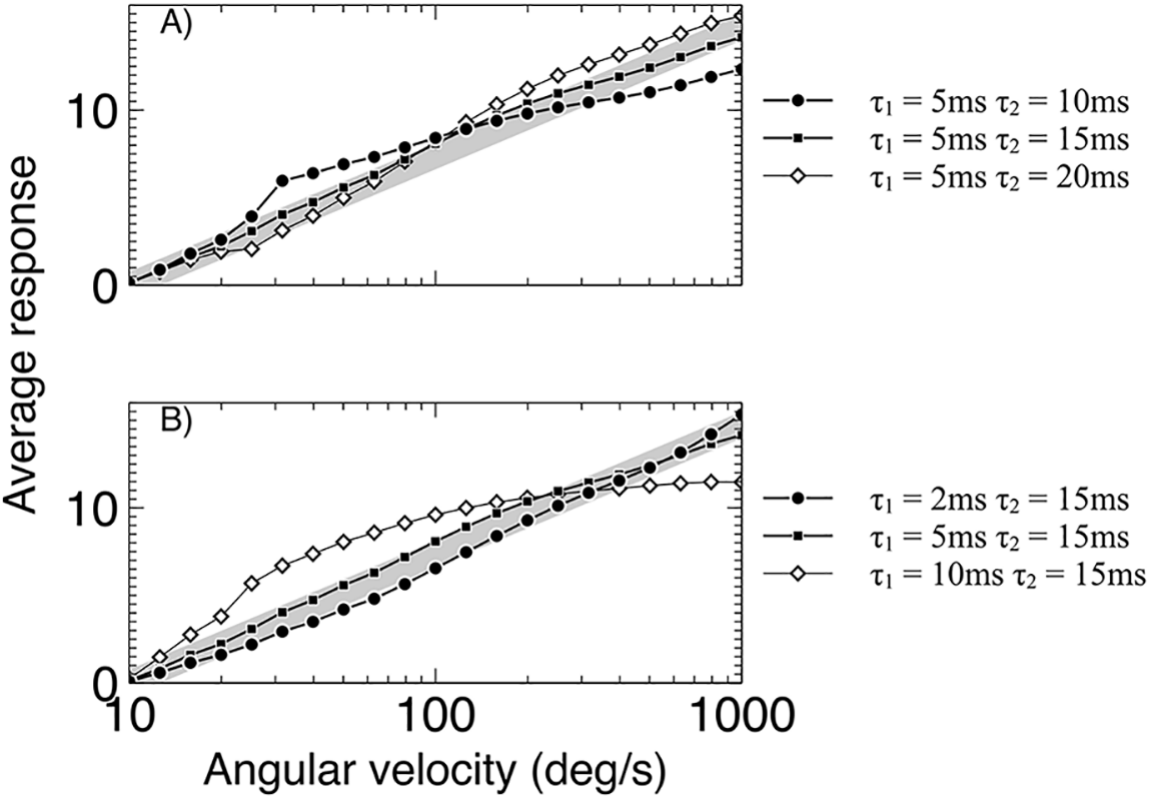
Comparison of the responses of dynamic time constant variants of the detector. (A) For a fixed time constant (τ_1_) and varying long time constant (τ_2_). (B) for a fixed long time constant (τ_2_) and varying short time constant (τ_1_). A best match to log-linear response for the 5/15ms time constant pair is found. All responses shown are for the detector responding to offsets only. The desired log-linear response is shown by the gray line.

To analyse the dynamical delays we investigated a range of pairs of values for *τ*_1_ and *τ*_2_, keeping one value fixed while varying the other; Fig 4 shows the results of this analysis. One possible source of the dynamical delay is the synaptic decay, which holds an exponentially decaying value of the input [45]. Different synaptic decay constants in the two different RHD-LIN detectors could lead to different responses. The model presented here does not allow us to definitively separate the synaptic decay and the membrane dynamics of the simple neural models used, as these are subsumed into the single global time constant for both rise and decay, however the difference in the two time constants used can give an indication of the possible neural mechanisms.

First we investigated the two limiting cases for pairs of detector delays qualitatively, assuming that the numerator of the division is always the response of the detector with the smaller delay (see Fig 2). In the first case the delays are identical. Thus the responses of the two detectors are almost the same (for small inter-ommatidial angles). In this case the detector response will asymptote towards a flat response across all frequencies. The second case is where the detector delays are vastly different. In this case the response of the detector with the smaller delay will be decreasing asymptotically towards zero as the second detector increases, leading to an exponential profile for the response of the whole model. We therefore expect that the responses will vary between flat and exponential depending on the ratio of the delays, with the absolute value of the delays determining the range of angular velocities over which this occurs.

In Fig 4 we see the responses of the model for different pairs of time constants. It is clear that the results follow our qualitative analysis, with the response approaching a flat response across all frequencies as the time constants become closer, and exponential as they separate. Nevertheless, all chosen value pairs produce an approximately log linear response profile as angular velocity increases, with the exception of the 10ms-15ms pair. This indicates a large stable range of time constant pairs which can still produce the responses found in the experimental data of Ibbotson [18]. The pair that in both cases is qualitatively closest to log-linear is the 5ms-15ms pair, therefore these are chosen as the best fit for the experimental data.

**Fig 5:**
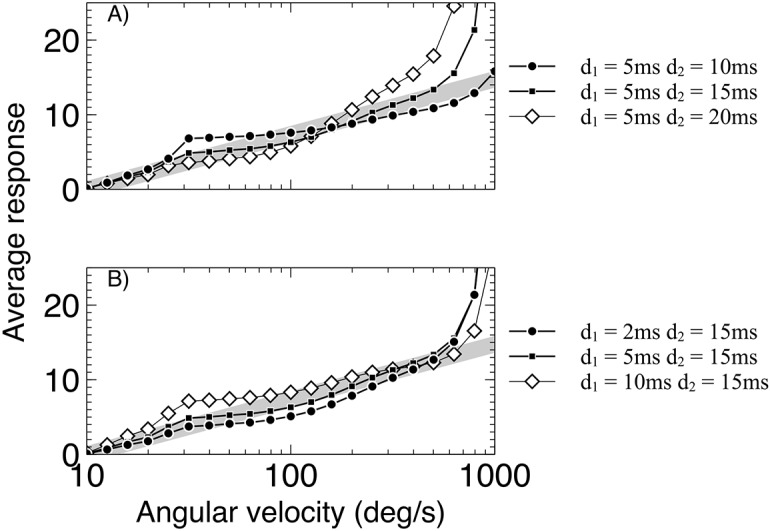
Comparison of the responses of fixed delay variants of the detector. (A) For a fixed short delay (d_1_) and varying long delays (d_2_). (B) For a fixed long delay (d_2_) and varying short delays (d_1_). A non-log linear response curve for all delay pairs is shown. All responses shown are for the detector responding to offsets only. The desired log-linear response is shown by the gray line.

The fixed delays were investigated using the same methodology as for the dynamical delays, and the results can be seen in Fig 5. In general the fixed delays do not show a log-linear response, but instead a linear or exponential response as angular velocity increases. This effect could be compensated by a non-linearity in the response curve of the neuron, so this alone does not discount the possibility that fixed delays could be used in the generation of angular velocity tuned responses. Two of the fixed delay pairs are notable: the 2ms-15ms pair shows a strong slope, which would be necessary for resolving accurately between angular velocities, although the response becomes very large at high values of angular velocity; the 10ms-15ms pair shows a long region that is approximately log-linear, but a weaker slope than the 2ms-15ms pair. Both of these will therefore be analysed further in the following section.

### Half rectification provides a better fit to the experimental data than no rectification or full rectification

The adaptive-LIN responds to increases and decreases in luminance with positive and negative output. While this is permissible for the photoreceptors and LMCs, which transmit sub-threshold information, the neurons following these in the column in the bee medulla transmit information as action potentials, and as such there is a biological constraint that their responses cannot transfer both positive and negative changes (i.e. onsets and offsets respectively). Therefore we shall test four methods of rectification of the adaptive-LIN output: the non-biological case of no rectification—where both onset and offset information are allowed to propagate (leaving the offset as a negative), half rectification where only onset information is propagated, half rectification where the offset information is propagated, and full rectification where all information is rectified and propagated. Of these these the half rectification is the most biologically plausible, as it can be implemented using either excitatory or inhibitory output from the LMC stage of processing.

**Fig 6:**
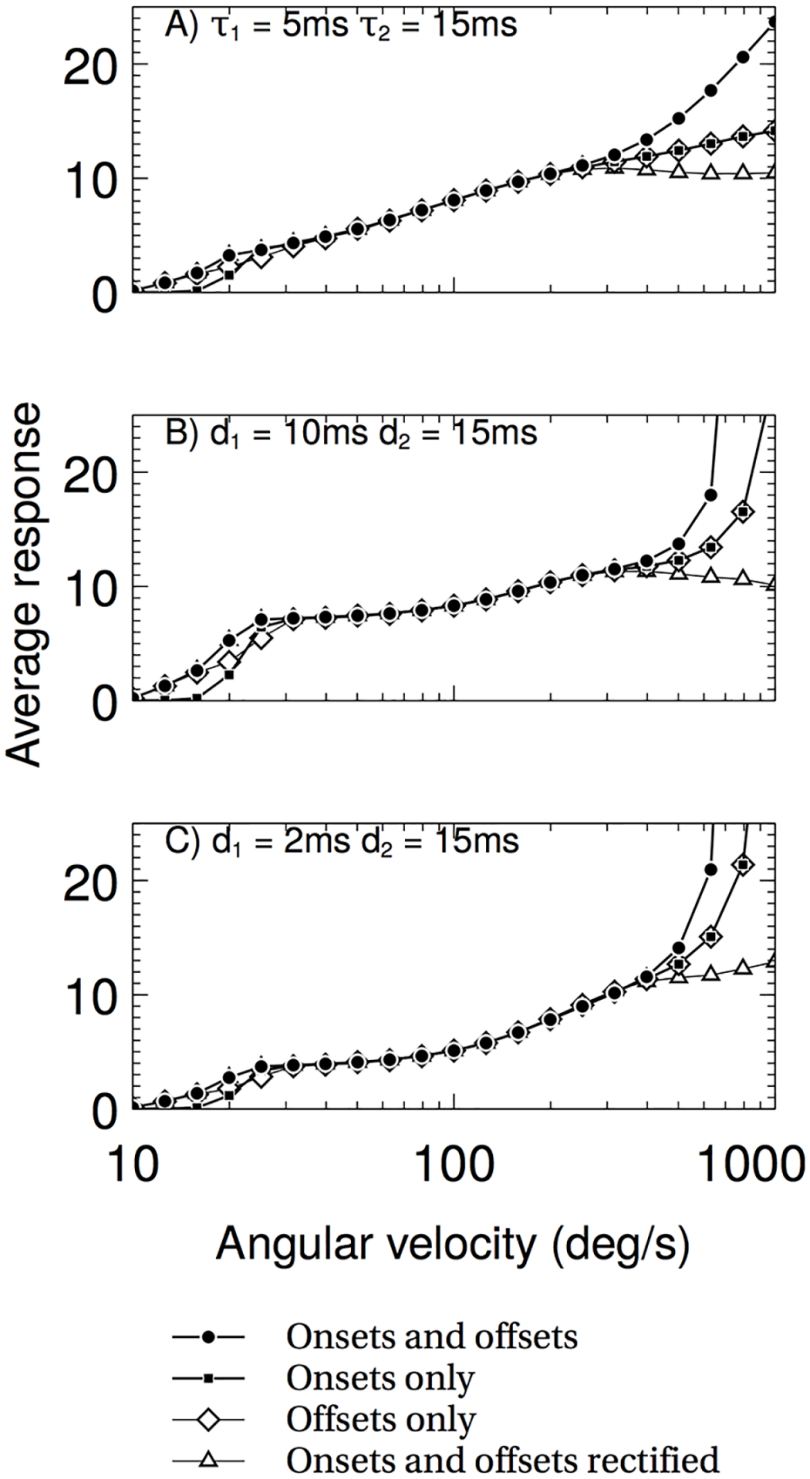
The average response of the full detector is largely invariant to the spatial frequency or contrast of the stimulus. The three plots show three different methods of the delay in the RHD-LIN detector, and the graphs within show the four different types of input. (A) a log-linear response is found for the onset-only and offset-only response curves. Both the rectified and non-rectified onset and offset response curves deviate from log-linear at high values of AV. (B) and (C) all response curves deviate from log-linear.

The detector pairs identified in the previous two analyses are investigated further here. Fig 6 shows the results; in all cases using onsets and offsets with no rectification causes a large increase in response at high angular velocities, and full rectification shows a decreased response. Aside from low angular velocities, the onset and offset half-rectified response curves are almost identical, and for the dynamical delay version of the model show a continued log-linear response that provides the best match to the experimental data. Given that recent evidence shows that onsets and offsets are processed in separate channels [15], it is interesting to note that such a mechanism provides the best fitting log-linear response from the angular velocity detector. For the purposes of this paper we will only model the offset channel as the responses of the two channels are extremely close.

It should be noted that a variant of the model using the Barlow-Levick elementary motion detector architecture was also tested. The results have been omitted as the detector both proved unsuitable for creating a velocity-tuned detector, and did not match the ordering of the responses to differing spatial frequency stimuli found in optomotor cells. Full details are provided in the Supporting Information (S1 Appendices).

### An AVDU with a log-linear response to increasing AV consists of RHD-LIN subunits with optomotor response properties

In the introduction we outlined the reasons why the classic optomotor neuron responses are unsuitable for generating corridor-centering behaviour. The neural circuitry underlying the optomotor response could, however, be utilised as part of the processing for an angular velocity tuned neuron. Having found the time constants and delays that produce a log-linear response to AV we now investigate if the RHD-LIN detectors comprising the AVDU match the properties of optomotor neurons in the honeybee.

**Fig 7:**
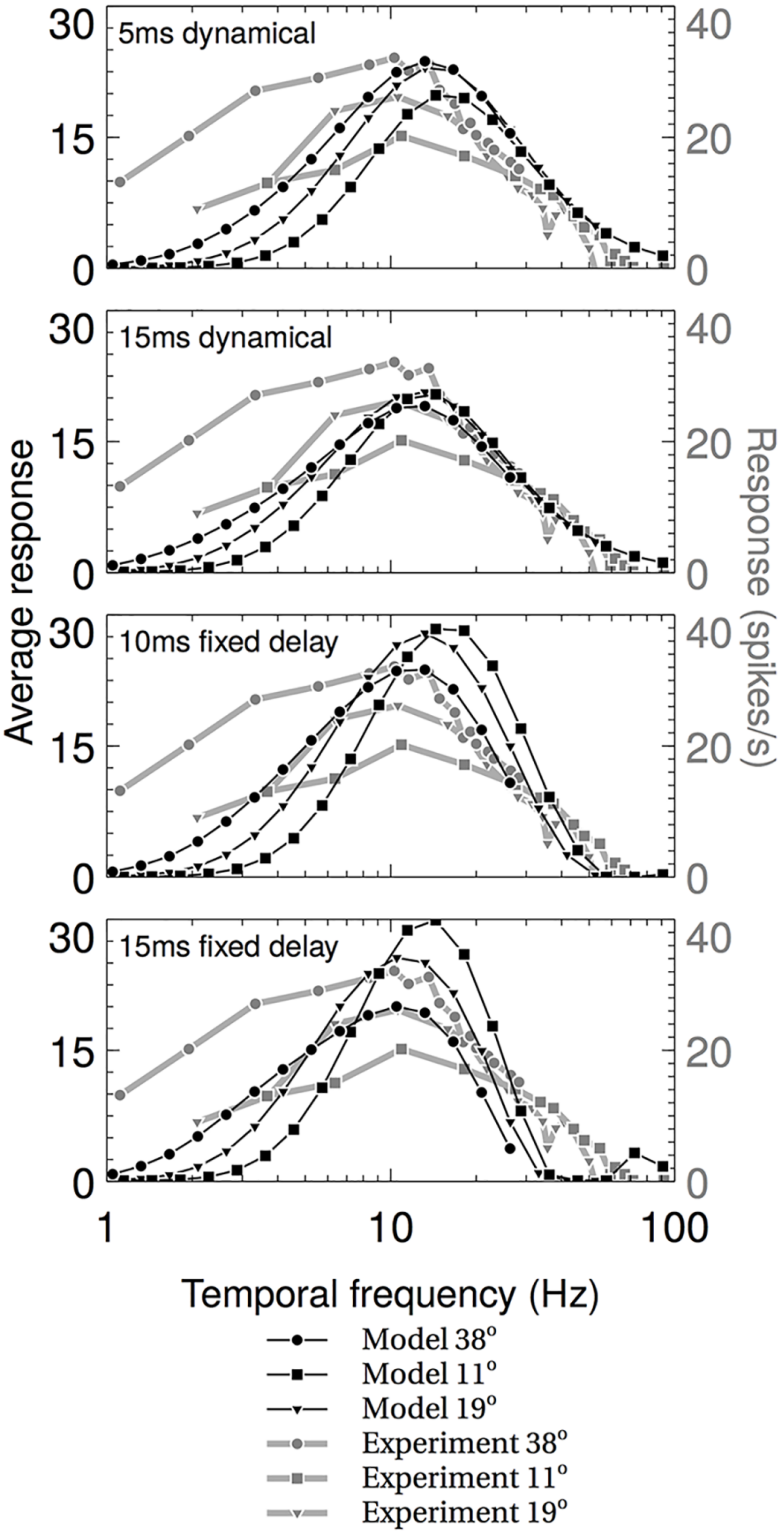
The average responses of the component RHD-LIN detectors for several spatial frequencies compared to experimental data of optomotor neurons. Experimental data is from Ibbotson 2001 [18]. The responses of the dynamical detectors (top two graphs) show a better match for the experimental data, notably the shared roll-off, than the fixed delay detectors.

The response of the RHD-LIN detectors to square wave gratings with spatial wavelengths of 11, 19 and 38 deg was tested by simulations using the Test system (see Methods) and extracting the RHD-LIN outputs before the division stage of the AVDU. Fig 7 shows the responses of the RHD-LIN detectors stimulated by three different spatial frequencies as the temporal frequency is varied in black. The results for the same experiment from an optomotor cell are shown in gray. The experimental response is characterised by a peak response around a temporal frequency of 10Hz for all spatial frequencies, and a roll-off that follows the same trajectory for all three spatial frequencies. There is also an ordering effect at low temporal frequencies and at the maximum responses, with the 38 degree stimulus having the highest response, and the 11 degree stimulus the lowest. The 5ms dynamical delay RHD-LINs reproduce all of these features, with the 15ms dynamical delay RHD-LIN missing the ordering at the peak; the fixed delay RHD-LINs reproduce the ordering only at low spatial frequencies, in addition to the ~10Hz maximum response. Interestingly, none of the model detectors reproduce the high response at low temporal frequencies that is found in the experimental data, suggesting that the neural implementation of the detector may be slightly different.

These results indicate that the optomotor neural circuitry would be a suitable building block in the creation of an AV-tuned response.

### The best fit AVDU response shows both spatial frequency and contrast invariance

**Fig 8:**
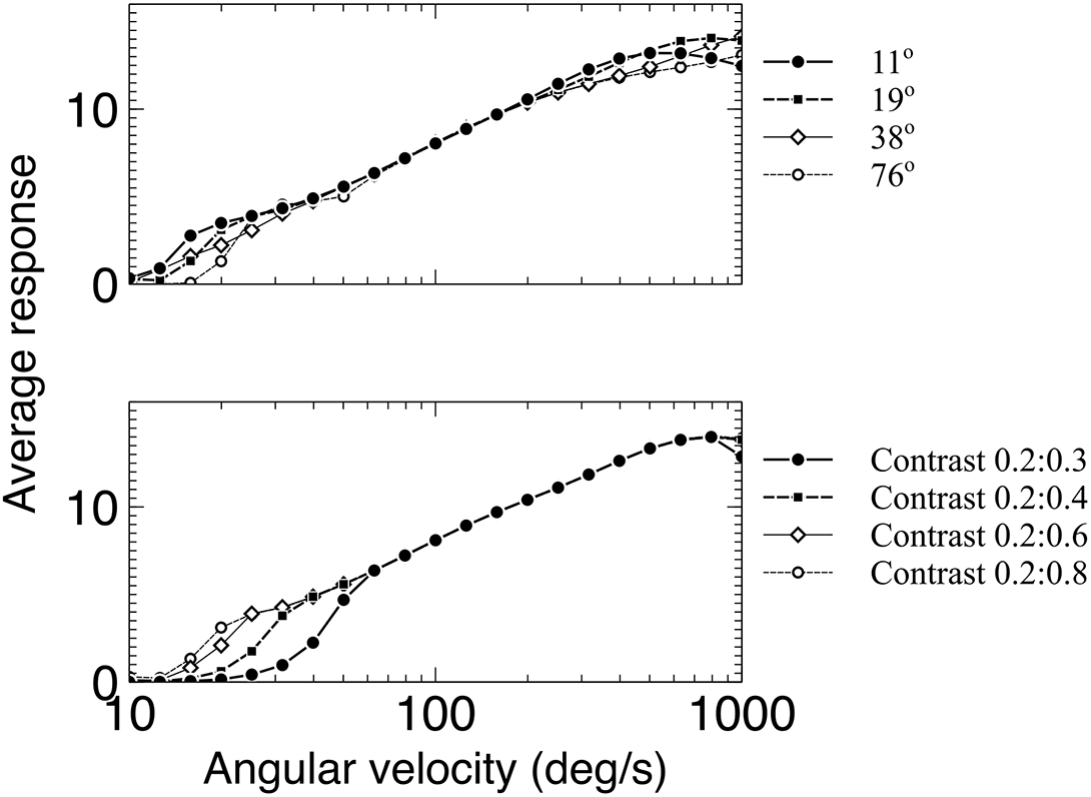
The average responses of the full detector to different spatial frequencies and different contrasts. The input has a spatial frequency of 19°, and the model *F* = 0.25. The response shows a clear velocity tuning that is largely invariant to the spatial frequency or contrast of the stimulus, with the exception of very low and high values of AV where there is greater variance.

The detector identified as most suitable by comparison to the responses of the velocity-tuned and optomotor neurons (5/15ms dynamic delay detector) was tested for invariance to changes in the spatial frequency and contrast of the stimulus, along with the direction of motion of the stimulus. If the detector is a candidate for reproducing the corridor-centering behaviour then it must show contrast and spatial frequency invariance and be directionally tuned as behavioural data exhibit this [1, 2]. Fig 8 shows the results of the experiments, demonstrating clear invariance over a range of angular velocities. Stimuli were all square wave gratings.

### The model accounts for the deviations from perfect corridor-centering in bee behaviour, and can be used as the basis for visual odometry

Here we make contact with the behavioural literature, which is especially important in modelling work on the bee as there is an abundance of behavioural experiments for honeybees and closely related species that can be used to test and add constraint to the model. We therefore simulate the full system (see Methods for details) embedded in a virtual environment, and the AVDU output is transformed into behaviour in the virtual environment using an established control algorithm adapted from Serres et al [46]. We have chosen results that primarily test the performance of the detector, rather than this control algorithm. The first experiment (Experiment 1A, see Methods) tests the limitations of the spatial frequency invariance when presented with narrow-band sinusoidal stimuli.

To compare our detector with the electrophysiological data we presented only square wave stimuli, as these are the stimuli that were used in the original experiments; there are no electrophysiological data on the performance of AV tuned neurons when presented with sinusoidal stimuli. To test the narrow-band performance of the detector we must therefore use behavioural experiments; we use the work of Dyhr et al [3], who investigated the corridor-centering accuracy of bumblebees when presented with a range of sinusoidal stimuli with different spatial frequencies.

**Fig 9:**
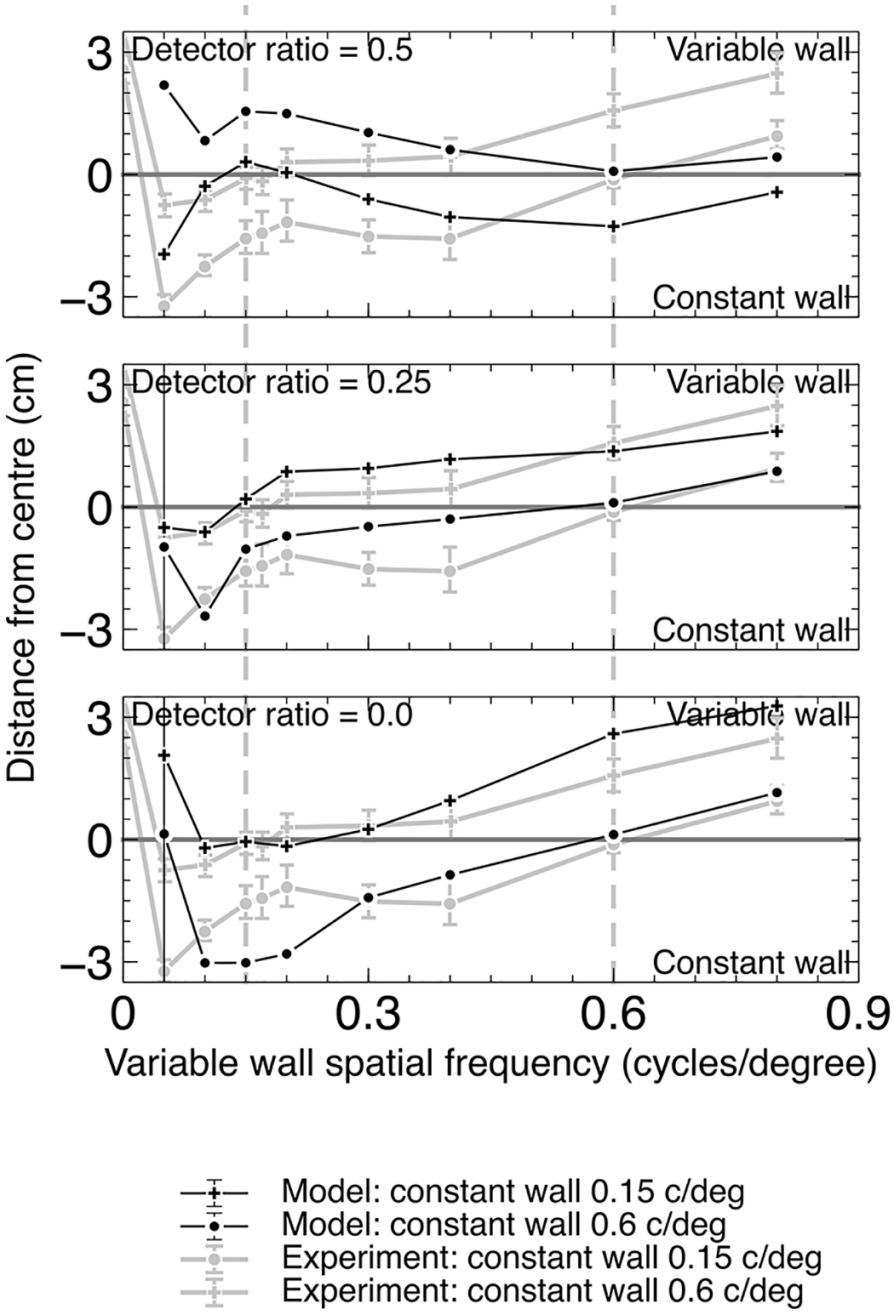
Centering performance of the model with *F* = 0.0 and *F* = 0.25 both agree with experimental data, while performance with *F* = 0.5 does not. Experimental data are from Dyhr et al [3] for real bees. One wall is held at a constant spatial frequency while the other is varied with sinusoidal patterns. Dashed lines indicate the two points where the spatial frequencies of the two walls are equal, one for each of the two lines. The model error bars show the variance of two runs with differing starting positions in the corridor.

Fig 9 shows the results of these experiments. The 0.5 ratio model does not match the experimental data, while the other two models both reproduce the qualitative pattern of deviations of the average flight path from the corridor centre observed in the data.

The second experiment we reproduced (Experiment 1B, see Methods) investigated the relationship between the wide-band (square wave) and narrow-band (sinusoidal) responses of the detector. This is an important test of the model, as this relationship was not tested for in selecting the detector.

**Fig 10:**
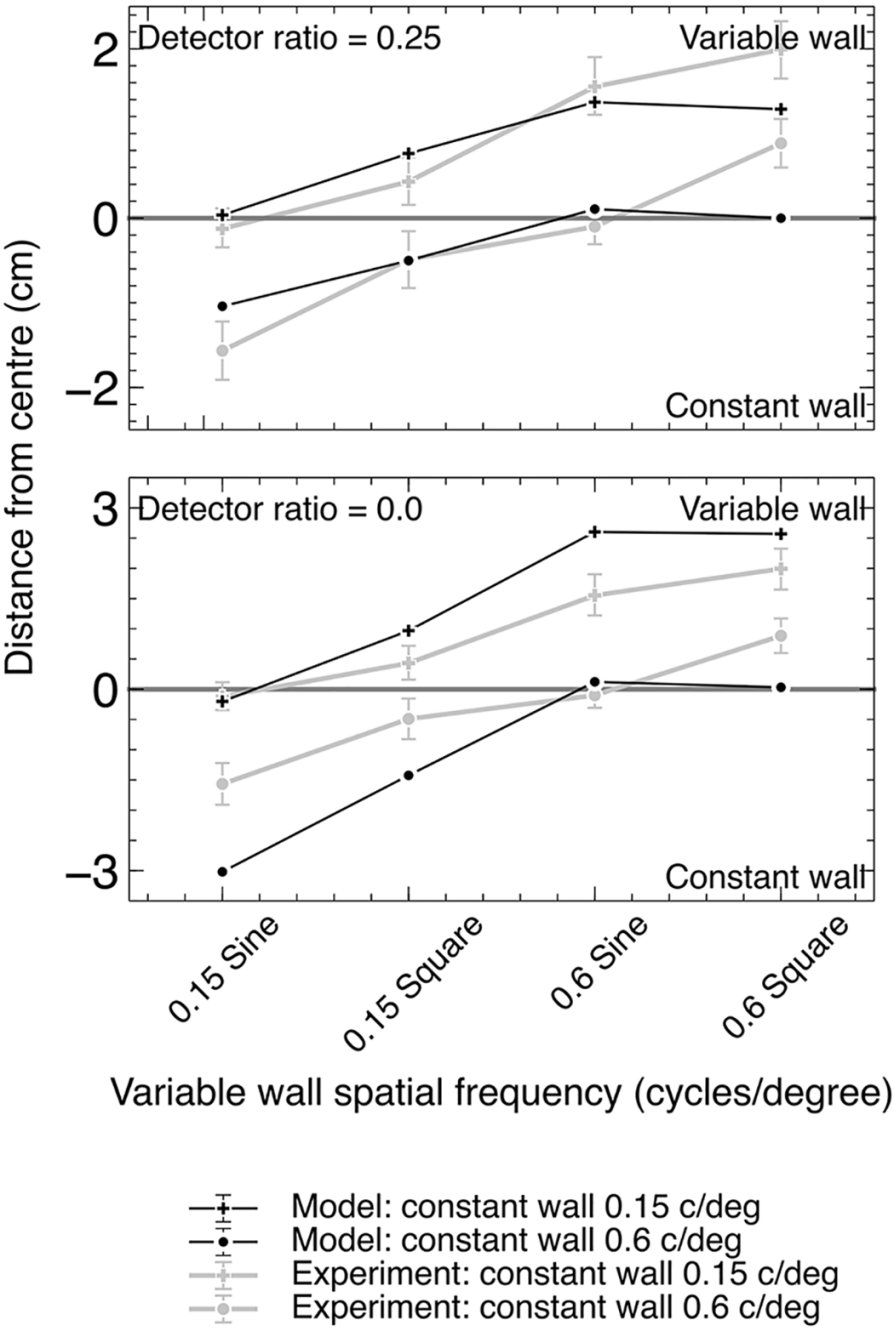
Centering performance of the model agrees with experimental data from real bees for *F* = 0.0 and 0.25. Experimental data is from Dyhr et al [3]. One wall was held at a constant spatial frequency while the other is varied, with square wave and sinusoidal patterns.

Fig 10 shows the results for the model with two values of *F*, compared with the experimental results. Both ratios qualitatively reproduce the pattern found in the experimental data, with the exception of the 0.6 c/deg square wave data.

**Fig 11:**
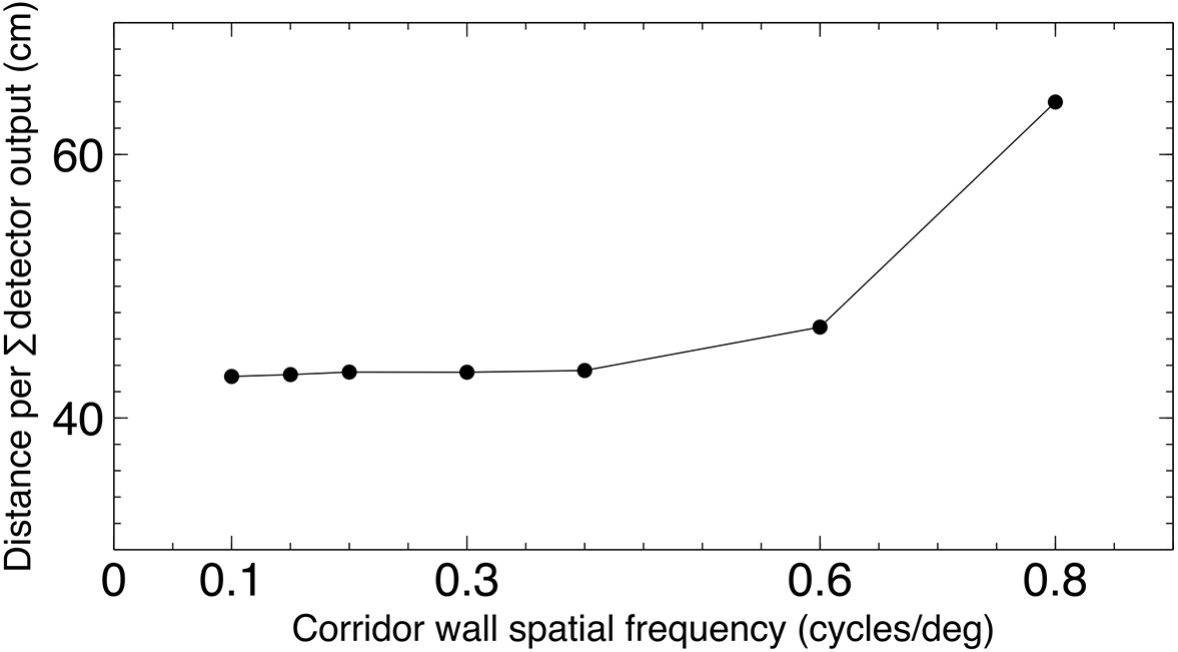
Odometry using the flight of the model bee. The simulated bee was flown in the for 5 seconds with the same stimuli on both walls. The stimuli were square wave gratings with spatial frequencies from 0.1 to 0.8 cycles per degree when observed from the corridor centre. Post-simulation the distance in cm per unit of the summed logged detector output is compared, and shows a consistent estimation of distance from the total summed detector output until 0.6 cycles per degree.

Experiment 2 (see Methods) investigated the suitability of the detector for odometry. The odometry that honeybees perform to find rewards is unaffected by the spatial frequency of the environment [2], and therefore it is possible that the AV tuned neuron responses could be used to estimate distance by summing the detector output over time and over all parts of the visual field. We tested the model to see if this relationship holds with our detector, and the results can be seen in Fig 11. The results show that for a wide range of spatial frequencies (0.1 to 0.6 c/deg) the detector presented here can provide a response that can be used for odometry by integrating the response over time.

## Methods

### Fitting experimental data with curves

Experimental data from Ibbotson [18] was extracted using the g3data tool and fitted with linear, logarithmic and exponential functions using the **lm** linear regression function in R [47] version 3.1.3. Errors introduced by use of the g3data tool are in all cases considerably less than 1% of the source plot axis length.

### Test system

The *Test System* consists of 200 input locations, corresponding to the AVDU‘photoreceptor’ stage, arranged into a 100x2 grid. AVDU detectors are oriented along the long axis, and there is one for each pair of locations, giving 99 AVDUs per row, and 198 total. All detectors have a preferred motion direction along the rows. The distance between the input locations corresponds to two degrees of angle in the *Beeworld* virtual environment (Supporting Information, S1 Appendices), given a 200x4 degree field of view.

All responses of the detectors to inputs were calculated by averaging the detector output for the final second of a two second simulation with this system.

### Full system

**Fig 12:**
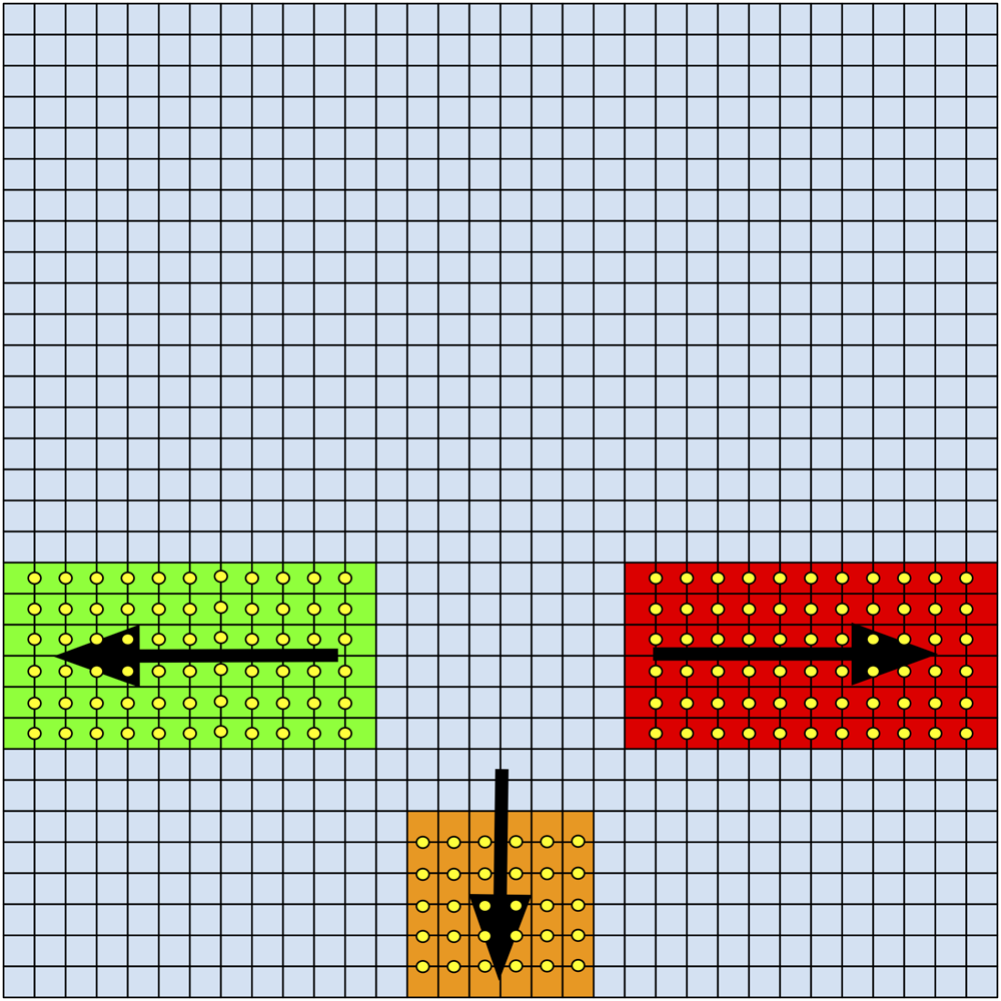
Layout of the full system showing subregions and AVDU placements. The input to the full system consists of 32x32 ommatidial locations (blue grid), which are processed by AVDUs in three subregions, left (green), right (red) and centre (orange). AVDUs (yellow circles) exist between the location pairs sharing the edge they are located on. The preferred motion direction of each subregion is shown with an arrow. Note that the 32x32 extent of the locations covers a field of view extending 260 degrees horizontally and 180 degrees vertically.

The *Full System* is designed to operate within a simulated corridor in our *Beeworld* virtual environment (see Supporting Information, S1 Appendices). The input consists of a 32x32 layer of simulated ommatidial locations, with detectors covering three subregions of this layer (left: rows 9–14 columns 1–12; right: rows 9–14 columns 20–32; centre: rows 1–6 columns 13–19) (see Fig 12). All subregions are sensitive to motion away from the centre of the layer, along the rows in the case of the left and right subregions, and the columns in the case of the bottom subregion. AVDUs cover each pair of locations (each location in the pair inputing into one of the ‘photoreceptors’ in one of the AVDU arms) along this motion sensitive direction, so there are 11 AVDUs per row for the left and right subregions, and 5 AVDUs per column for the centre subregion. These subregions contribute to single individual summation LINs (denoted by *τ*_*s*_ in Fig 2) (i.e. three LINs total, one for each subregion), and the response of these separate summation LINs is used to guide the virtual honeybee by input into a flight controller. These summed outputs will be refered to as S_L_, S_R_ and S_C_ for the left sum, right sum and centre sum respectively. The field of view of the model is set to 260 degrees horizontally and 180 degrees vertically, providing approximately 1/5 the resolution of the honeybee eye. As a controller we use that proposed by Serres et al [46, 48]. The controller is configured using setpoints for the horizontal, vertical and velocity controllers (respectively T_X_ = 1.0, T_Z_ = 0.5 and T_V_ = 2T_H_). Scalings are then applied to change the controller outputs into changes for one timestep to the horizontal position X, vertical position Z and velocity V. Thus the equations for generating changes in the simulated bee’s flight are:

$$\Delta X=sgn\left(S_{L},S_{R}\right)\left(max\left(S_{L},S_{R}\right)-T_{X}\right))/2.4$$

$$\Delta Z=(S_{C}-T_{Z})/18.0$$

$$\Delta V=-(S_{L}+S_{R}-T_{V})/2.4$$

Where *sgn*(*A*,*B*) is positive for *A* > *B* and negative for *A* < *B*, and *max*(*A*,*B*) is the maximum value out of *A* and *B*.

### Fitting the model to electrophysiological data

The model is tuned using the Test system. Square wave input is used as this is the input used in the electrophysiological experiments [18], ranging from 10 deg/s to 1000 deg/s and with a wavelength of 38 degrees.

### Virtual behavioural experiments

It is important to verify the performance of the model against behavioural data. For this we choose two experimental paradigms which require AV estimation to perform and compare our simulated behaviour with data from the literature taken from ethological experiments performed in the field with real bees. In these cases to reproduce the data we must substitute the real world visual input to the real bees with virtual visual data input into our model. This is provided by a ray-traced environment (Beeworld, see Supporting Information: S1 Appendices).

**Experiment 1: The *corridor-centering paradigm*.** Bees were trained to repeatedly seek a reward at one end of a corridor, with the entrance at the opposite end [1–3, 29, 49]. The bees were able to centre themselves laterally in the corridor with only small deviations, regardless of differences in the spatial frequency components of the patterns of the two corridor walls. The ability to centre by itself is not of interest to testing the model, as any detector of AV (including perfect detectors) will show this behaviour. Instead it is the deviations from perfect centering that we will use to validate the model. We reproduce the magnitude and pattern of deviations as the spatial frequencies on the two corridor walls are varied, as well as the deviations due to the use of square wave and sinusoidal grating stimuli. The details of these two experiments are given below.

It should be noted that all spatial frequencies described here are measured by the angular frequency the bee would observe from the centre of the corridor, and since the spatial resolution of our model is approximately five times lower than that of the bee we present spatial frequencies five times smaller. For all these experiments the Full system was used.

**Experiment 1A.** Honeybees and bumblebees show a small tendency for the average flight path to move towards a wall with a higher spatial frequency (e.g. up to 3cm in a 20cm wide corridor) [3]. In the first experiment we reproduced, one wall was fixed at a spatial frequency of 0.15 or 0.6 cycles per degree (c/deg) (0.03 or 0.12 c/deg for the model), while spatial frequencies ranging from 0.05 to 0.8 were presented on the other wall. These walls are termed the *constant wall* and the *variable wall* respectively. We used three different ratio levels for the RHD-LIN detectors (0.0, 0.25, and 0.5). The model is deterministic, but two repetitions of the model from different starting locations (3cm either side of the corridor centre at 6cm height and 40 cm/s starting speed) are used. These two repetitions account for possible biases induced by the starting position. Centering behaviour was consistent within each pair of runs, except for the 0.12 c/deg pattern which has the least motion information. The model was run for five seconds and the final position value was determined by averaging over the final two seconds of flight.

**Experiment 1B.** Bees exhibit different deviations when square and sinusoidal gratings with equal spatial frequencies are used. In these experiments one wall had a narrow-band pattern, and the other wall either a narrow-band or wide-band pattern [3]. Two spatial frequencies (0.15 and 0.6 c/deg (0.03 and 0.12 c/deg for the model)) were used for both walls, giving eight combinations of pattern type and spatial frequency. The model was run for five seconds and the final position value is determined by averaging over the final two seconds of flight.

**Experiment 2: The bee visual odometer.** This is used to calculate the distance bees have flown and permit returning to a location a fixed distance from the hive. The odometer allows bees to return to a fixed, rewarded, location in a corridor even when the spatial frequencies of the patterns on the corridor walls are changed. If the tunnel carries no texture that can provide optic flow cues, the bees cannot accurately return to the rewarded location [2]. This behaviour also requires the calculation of the AV of the environment, without which the spatial frequency of the walls would distort the distance recorded by the odometer. A mechanism for integrating the AV signals over time is also required, however we do not explicitly model this here as such a mechanism would be required for any distance mesasurement circuit.

To test the ability of the model to reproduce this behaviour the model bee was flown for 5 seconds with the same stimuli on both walls. The stimuli were square wave gratings with spatial frequencies from 0.1 to 0.8 cycles per degree when observed from the corridor centre. Estimated distance from post-simulation integration of the logged model output was then compared with distance flown by the virtual bee, which was extracted from the simulation environment. As the speed of the bee can vary, the summed output is standardised by dividing by the total distance travelled by the virtual bee, to give the summed output per unit distance. The following equation describes the metric used:

$$D_{E}=\frac{\sum_{t=0}^{5}\left(S_{L}\left(t\right)+S_{R}\left(t\right)\right)}{D}$$

where *D*_*E*_ is distance estimate—the summed detector output per unit of distance travelled in the virtual environment, *S*_*L*_ and *S*_*R*_ are the summed AVDU detector array outputs at time *t* for the left and right detector regions in the Full model, and *D* is the total distance travelled in the virtual environment over the 5 seconds of simulation.

### Simulation

The model, both in the form of the Test system and the Full system, is described in SpineML and is simulated using the SpineML 2 BRAHMS code generation [50]. Code for the model can be found at http://greenbrain.group.shef.ac.uk/research/vision/. Communication between Beeworld and the model is performed using SpineML TCP/IP interfaces through localhost. All simulations are performed using a timestep of 0.1ms.

## Discussion

We have presented a model, the Angular Velocity Detector Unit (AVDU), that reproduces several behavioural patterns including previously unaccounted for observations in the bee corridor-centering response. These are: the deviation of the average flight path from the corridor centre according to differences in spatial frequency of sinusoidal gratings on opposite walls, and the deviations of the average flight path from the corridor centre for square wave and sinusoidal gratings. In addition the AVDU can be used as the basis of the experimentally confirmed bee visual odometer, allowing bees to gauge distance by integrating the output from the detector while navigating to a rewarded location. The model is based on the neural circuits found in the insect brain and proposes a mechanism that can bridge the gap between the retinotopic elementary motion detection circuits in the visual neuropils [15–17] and the wide-field AV estimating neurons in the ventral nerve cord [18] with minimal additional neural circuitry.

Before discussing the behaviour of the model in detail we first consider the response of the AV estimating neurons recorded by Ibbotson [18]. All neurons showed a monotonically increasing response to angular velocity which was largely insensitive to spatial frequency, however there is large variation between the functions that best fit the data, ranging from log-linear through to exponential. Interestingly, we find that as the parameters of the model are changed to fit the data, a similar variation in the form of the response can be observed. One possible explanation for these results is that the AV estimating neurons collectively constitute an ensemble estimator, which is capable of more accurate AV estimation due to the steeper response gradients of different neurons being combined selectively to produce motor responses. Such a scheme would predict a range of Reichardt-Hassenstein detectors with different delays or detector spacings (e.g. taking the correlation of every two ommatidial spacing rather than every one) in the insect optic neuropils.

We adopted a two stage process to validate the AVDU; first we tuned the free model parameters based upon the response of the AV-sensitive descending neurons, then we tested the AVDU against both electrophysiological data of the optomotor response and behavioural data. By separating the tuning and testing stages we avoided overfitting the AVDU to the data; it is not necessary that an AVDU that performs well in our fitting stage will also match the data in the testing stage.

Patterns in the behavioural data from real bees are diagnostic of possible underlying mechanisms. In the corridor-centering task a perfect AV detector would be completely insensitive to the spatial frequency of the stimuli on the corridor walls. Therefore the mean horizontal flight position in the corridor would be expected to be the centre, even with different spatial frequencies on the corridor walls. An imperfect detector, however, will be offset from the centre except in the cases where the two walls have the same spatial frequency. The direction and magnitude of these offsets will depend on the differential response of the detector to different spatial frequencies, and it is not clear *a priori* what the offsets should be. Having tuned our detector to match idealised electrophysiological data it could not be directly expected from the tuning procedure that the AVDU would reproduce the offsets found experimentally, especially considering that the AVDU is tuned with broadband square-wave gratings and tested with narrowband sinusoidal gratings. We find that the model does, however, reproduce the pattern of offsets found behaviourally, excepting the lowest spatial frequencies (a deficit due to the fivefold reduction in receptors in the model test system compared to a bee). The model also describes the relative offsets between square-wave and sinusoidal stimuli, which again could not be expected from the tuning procedure.

Our model operates by combining the outputs from Reichardt-Hassenstein Detectors (RHD) with different delay time constants to produce a response that estimates the AV of motion of the environment. These are chosen as the Reichardt-Hassenstein circuitry has been well validated as the basis of elementary motion detection in the insect optic neuropils in *Drosophila melanogaster* [15–17]. The output of each RHD is consistent with the response profile of the optomotor response. This allows the AV response to be generated by selectively sampling from a pool of RHD detectors with different time delays. We therefore propose that the AV response, along with other motion sensitive responses in the bee, arises from an evolutionarily conserved set of elementary motion detectors. The advantages of such an organisational structure are twofold. First, the optic neuropils of the bee consist of a highly columnar structure from the photoreceptors through to the first layers of the lobula, followed by a set of wide field neurons that extract information from across the visual field [19–23, 51]. Adding additional columnar elements throughout the lamina, medulla and lobula would require extensive changes to the optic neuropils. Conversely, adding neurons to combine the outputs of the relatively few wide field neurons would not require such extensive changes, and is more conservative from an evolutionary perspective. Second, the energy requirements of the neurons in a separate channel through columnar parts of the optic neuropils would be high (the energy cost of the photoreceptors and LMCs alone in the blowfly are estimated to consume 10% of the total required resting energy [30]), requiring significant evolutionary benefit to fitness for the investment [31]. Our model therefore has fewer retinotopic information channels in the optic neuropils (as the same information channels are used by the optomotor and AV estimating systems) and given the metabolically cost of information transfer [30, 32–34] is less energetically costly than separate retinotopic AV and optomotor processing. Although this may not provide as robust a measure of AV as may be possible with dedicated neural circuits, we suggest that the energetic advantages could outweigh any disadvantages.

## Supporting Information

S1 Appendix Beeworld, Analysis of neuron responses and Barlow-Levick detector results. A description of the Beeworld raytracer used to provide input to the models and the results of assessing the performance of the Barlow-Levick form of coincidence detector in place of the Reichardt-Hassenstein detectors.
(PDF)
