# Supplementary material for: A Model for an Angular Velocity-Tuned Motion Detector Accounting for Deviations in the Corridor-Centering Response of the Bee
Source: PLoS Comput Biol. 2016 May 5;12(5):e1004887. doi: 10.1371/journal.pcbi.1004887 (PMC4858260; doi:10.1371/journal.pcbi.1004887)
Supplement: S1 Appendix — A description of the Beeworld raytracer used to provide input to the models and the results of assessing the performance of the Barlow-Levick form of coincidence detector in place of the Reichardt-Hassenstein detectors. (PDF) [file pcbi.1004887.s001.pdf]

Appendices

December 31, 2015

Part I
Beeworld

Stimuli to the model are provided by means of a virtual environment called Beeworld. This environment is instantiated using an XML description denoting three types of primitive object (planes, cylinders and spheres) along with procedural textures which can be attached to these primitives (currently checked and radial stripes). Instances of these primitives and textures can be parameterised to control their location, size, rotation, spatial frequency and motion. Included in the XML is a description of the location and attitude of the camera representing the bee, along with the field of view and resolution. A custom raytracer is used to render this XML description into an array of trichromatic colour values for input into the model. The raytracer casts a ray for each ommatidium of the simulated bee. An example of an XML world is shown below, and is the world used for the test system described later. A view from Beeworld can be seen in Figure 1.

```
<BeeworldConfig>
    <System>
        <NumConnections value="2"/>
        <DisplayScaling value="1.0"/>
        <Blur value="false"/>
        <Lighting value="false"/>
        <Log path=""/>
        <DT value="0.1"/>
    </System>
    <BeeEye>
        <Vertical FOV="2" Resolution="2"/>
        <Horizontal FOV="200" Resolution="100"/>
    </BeeEye>
    <BeeInitialState>
        <Time value="0"/>
        <X value="0"/>
        <Y value="0"/>
        <Z value="3"/>
        <Roll value="0"/>
        <Pitch value="0"/>
        <Yaw value="0"/>
        <Speed value="0"/>
        <TransverseSpeed value="0"/>
    </BeeInitialState>
    <Controllers>
```

```
        <Controller source="freq" target="r1" port="RadialMotion"/>
    </Controllers>
    <World>
        <Cylinder name="cy1">
            <Location x="0" y="0" z="0"/>
            <Rotation xAxis="0" yAxis="0" zAxis="0"/>
            <Scaling x="2" y="2" z="6"/>
            <Texture>
                <Radial name="r1">
                    <Frequency value="9.48"/>
                    <RadialOffset value="0.0"/>
                    <RadialMotion value="90.0"/>
                    <Offset x="0.0" y="0.0" z="0.0"/>
                    <Motion x="0" y="0" z="0"/>
                    <LightCol r="0.8" g="0.8" b="0.8"/>
                    <DarkCol r="0.2" g="0.2" b="0.2"/>
                </Radial>
            </Texture>
        </Cylinder>
    </World>
</BeeworldConfig>
```

Two environments are used in the experiments described here. In the first the bee is centred in a cylinder with a radius of 1cm. The cylinder is oriented with the circular axis in the z-direction. It has a texture consisting of vertical radial stripes with a spatial period of 11, 19, 38 or 76 degrees and motion directed around the vertical z-axis, the angular velocity of which can be varied to test the velocity tuning of the detectors. The bee is oriented along the y-axis and can be rotated about this axis to change the apparent direction of the motion stimuli (to test directional preference of the detectors).

The second experimental environment is a corridor configured to the specifications used by Dyhr et al[1], with the angular frequencies adjusted to compensate for the lower resolution of the model than the honeybee. The walls are textured with sinusoidal or square wave gratings oriented vertically, whose spatial frequency and contrast can be varied. This environment allows behavioural experimental tests of the detector performance in combination with an established control algorithm [2].

Part II
Analysis of neuron responses

Table 1 shows the adjusted R^2 values for fittings of different functions to the velocity tuned detector response data from [3]. Logarithmic fits show the greatest adjusted R^2 value in the majority of cases.

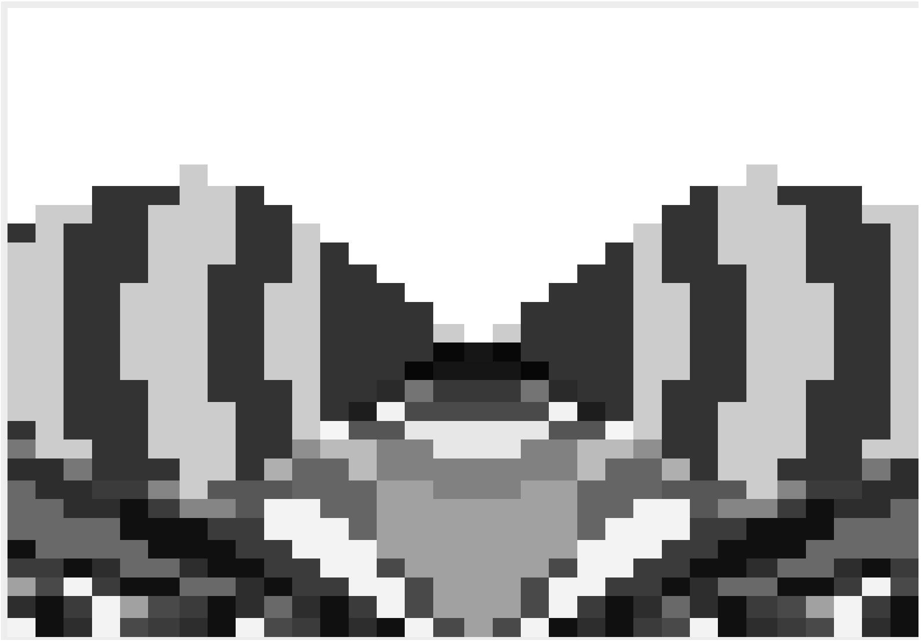

Figure 1: A view down a corridor in Beeworld

	Logarithmic fit adjusted R^2	Linear fit adjusted R^2	Exponential fit adjusted R^2
11° stimulus	**0.7821**	0.4905	0.5139
	**0.8976**	0.8483	0.8101
19° stimulus	**0.8561**	0.6893	0.2177
	**0.7536**	0.6004	0.5936
	**0.7387**	0.5509	0.5115
	0.3167	0.3824	**0.4055**
	0.6288	0.7623	**0.7665**
38° stimulus	**0.9213**	0.8594	0.6549
76° stimulus	**0.8925**	0.7223	0.6012
	**0.9318**	0.8474	0.7806
	0.7154	0.7774	**0.8449**
	0.7141	**0.8725**	0.8636
	0.4993	0.5855	**0.6816**
Average	**0.7422**	0.6914	0.6343

Table 1: Adjusted R^2 for various fittings of the data from Ibbotson 2001. Best fits are marked in bold text.

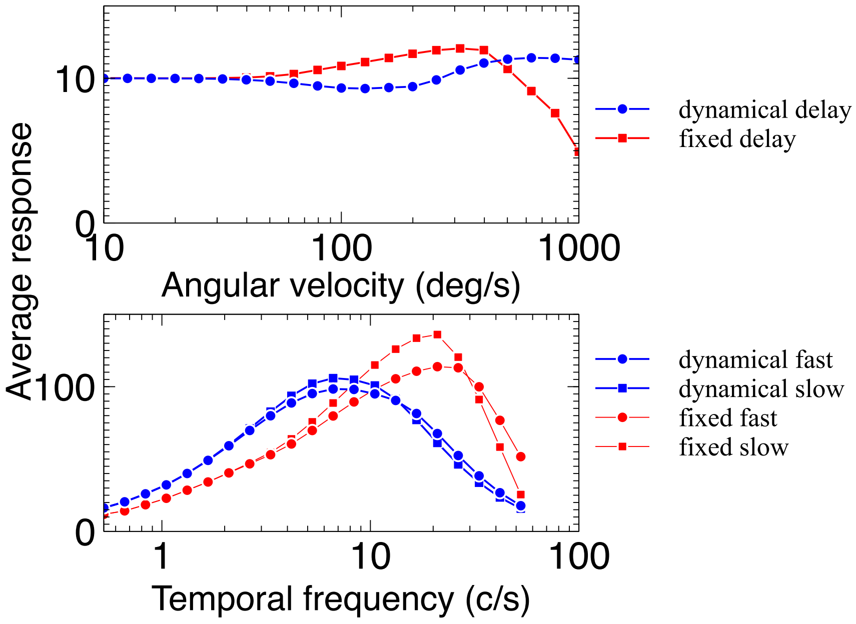

Figure 2: Barlow-Levick detector. results for a detector with time constants of 5ms and 15ms (blue), or fixed delays of 5ms and 15ms (red). In both cases there is little difference in the ratio of the two RHD-LIN detectors (bottom plot), leading to flat responses of the full AVDU (top plot). These results are characteristic of those found with other time constants.

Part III

Barlow-Levick detector results

Result for an example Barlow-Levick detector based AVDU can be seen in Figure 2.

References

[1] Dyhr JP, Higgins CM (2010) The spatial frequency tuning of optic-flow-dependent behaviors in the bumblebee Bombus impatiens. The Journal of Experimental Biology 213: 1643–50.

[2] Portelli G, Serres J, Ruffier F, Franceschini N (2010) Modelling honeybee visual guidance in a 3-D environment. Journal of Physiology 104: 27–39.

[3] Ibbotson MR (2001) Evidence for velocity-tuned motion-sensitive descending neurons in the honeybee. Proceedings Biological sciences / The Royal Society 268: 2195–201.
